# HIAYA CHAT study protocol: a randomized controlled trial of a health insurance education intervention for newly diagnosed adolescent and young adult cancer patients

**DOI:** 10.1186/s13063-022-06590-5

**Published:** 2022-08-19

**Authors:** Karely Mann, Austin R. Waters, Elyse R. Park, Giselle K. Perez, Perla L. Vaca Lopez, Heydon K. Kaddas, Echo L. Warner, Nicole Ray, Tomoko Tsukamoto, Karlie Allen, Ben Haaland, Douglas B. Fair, Mark A. Lewis, Anne C. Kirchhoff

**Affiliations:** 1 Cancer Control & Population Sciences, Huntsman Cancer Institute, University of Utah, Salt Lake City, UT USA; 2 Department of Health Policy & Management, Gillings School of Global Public Health, University of North Carolina Chapel Hill, Chapel Hill, NC USA; 3 Health Promotion & Resiliency Intervention Research Program, Mongan Institute, Boston, MA USA; 4 Departments of Psychiatry & Medicine, Mass General Hospital/Harvard Medical School, Boston, MA USA; 5 College of Nursing, University of Utah, Salt Lake City, UT USA; 6 Adolescent and Young Adult Cancer Care Program, Intermountain Healthcare, Salt Lake City, UT USA; 7 AYA Patient Navigation Program, Huntsman Cancer Hospital, Salt Lake City, UT USA; 8 Department of Population Health Sciences, University of Utah, Salt Lake City, UT USA; 9 Huntsman Cancer Institute, University of Utah, Salt Lake City, UT USA; 10 Department of Pediatrics, Division of Pediatric Hematology/Oncology, University of Utah, Salt Lake City, UT USA; 11 Primary Children’s Hospital, Intermountain Healthcare, Salt Lake City, UT USA

**Keywords:** Adolescent and young adult, Cancer, Pilot randomized controlled trial, Health insurance, Health insurance literacy, Patient navigation, Virtual intervention

## Abstract

**Background:**

For adolescent and young adult (AYA) cancer patients aged 18 to 39 years, health insurance literacy is crucial for an effective use of the health care system. AYAs often face high out-of-pocket costs or have unmet health care needs due to costs. Improving health insurance literacy could help AYAs obtain appropriate and affordable health care. This protocol illustrates a randomized controlled trial testing a virtual health insurance education intervention among AYA patients.

**Methods:**

This is a two-arm multisite randomized controlled trial. A total of 80 AYAs diagnosed with cancer in the Mountain West region will be allocated to either usual navigation care or tailored health insurance education intervention with a patient navigator that includes usual care. All participants will complete a baseline and follow-up survey 5 months apart. The primary outcomes are feasibility (number enrolled and number of sessions completed) and acceptability (5-point scale on survey measuring satisfaction of the intervention). The secondary outcomes are preliminary efficacy measured by the Health Insurance Literacy Measure and the COmprehensive Score for financial Toxicity.

**Discussion:**

This trial makes a timely contribution to test the feasibility and acceptability of a virtual AYA-centered health insurance education program.

**Trial registration:**

ClinicalTrials.gov NCT04448678. Registered on June 26, 2020

## Administrative information

Note: the numbers in curly brackets in this protocol refer to SPIRIT checklist item numbers. The order of the items has been modified to group similar items (see http://www.equator-network.org/reporting-guidelines/spirit-2013-statement-defining-standard-protocol-items-for-clinical-trials/).

**Table:** 

Title {1}	HIAYA CHAT study protocol: a randomized controlled trial of a health insurance education intervention for newly diagnosed adolescent and young adult cancer patients
Trial registration {2a and 2b}.	ClinicalTrials.gov ID: NCT04448678. Item 2b is not applicable for this protocol.
Protocol version {3}	Protocol version 4. Date released: 8/10/2021
Funding {4}	U.S. NIH Grant/Contract Award Number: 1R01CA242729-01
Author details {5a}	Karely Mann • Cancer Control & Population Sciences, Huntsman Cancer Institute, University of Utah, Salt Lake City, Utah Austin R. Waters • Cancer Control & Population Sciences, Huntsman Cancer Institute, University of Utah, Salt Lake City, Utah • Department of Health Policy & Management, Gillings School of Global Public Health, University of North Carolina Chapel Hill, Chapel Hill, NC Elyse R. Park • Health Promotion & Resiliency Intervention Research Program, Mongan Institute, Boston, Massachusetts • Departments of Psychiatry & Medicine, Mass General Hospital/Harvard Medical School, Boston, Massachusetts Giselle K. Perez • Health Promotion & Resiliency Intervention Research Program, Mongan Institute, Boston, Massachusetts • Departments of Psychiatry & Medicine, Mass General Hospital/Harvard Medical School, Boston, Massachusetts Perla L. Vaca Lopez • Cancer Control & Population Sciences, Huntsman Cancer Institute, University of Utah, Salt Lake City, Utah Heydon K. Kaddas • Cancer Control & Population Sciences, Huntsman Cancer Institute, University of Utah, Salt Lake City, Utah Echo L. Warner • College of Nursing, University of Utah, Salt Lake City, Utah • Cancer Control & Population Sciences, Huntsman Cancer Institute, University of Utah, Salt Lake City, Utah Nicole Ray • Cancer Control & Population Sciences, Huntsman Cancer Institute, University of Utah, Salt Lake City, Utah Tomoko Tsukamoto • Adolescent and Young Adult Cancer Care Program, Intermountain Healthcare, Salt Lake City, Utah Karlie Allen • AYA Patient Navigation Program, Huntsman Cancer Hospital, Salt Lake City, Utah Ben Haaland • Department of Population Health Sciences, University of Utah, Salt Lake City, Utah • Cancer Control & Population Sciences, Huntsman Cancer Institute, University of Utah, Salt Lake City, Utah Douglas B. Fair • Huntsman Cancer Institute, University of Utah, Salt Lake City, Utah • Department of Pediatrics, Division of Pediatric Hematology/Oncology, University of Utah, Salt Lake City, Utah • Primary Children’s Hospital, Intermountain Healthcare, Salt Lake City, Utah Mark A. Lewis • Adolescent and Young Adult Cancer Care Program, Intermountain Healthcare, Salt Lake City, UT Anne C. Kirchhoff • Cancer Control & Population Sciences, Huntsman Cancer Institute, University of Utah, Salt Lake City, Utah • Department of Pediatrics, Division of Pediatric Hematology/Oncology, University of Utah, Salt Lake City, Utah
Name and contact information for the trial sponsor {5b}	University of Utah, 201 Presidents’ Circle, Salt Lake City, UT 84112, 801-581-7200
Role of sponsor {5c}	Not applicable. The University of Utah takes no role in study design or management.

## Introduction

### Background and rationale {6a}

Quality health insurance coverage is critical to improving health outcomes for cancer patients diagnosed as adolescents and young adults (AYAs) [1, 2]. Health insurance can be complex to obtain for many AYA cancer patients, especially for those who have aged out of dependent health insurance coverage [3, 4]. Managing insurance and finances during treatment is a source of stress and confusion for AYA cancer patients [5–7]. AYAs diagnosed with cancer are often underinsured and face high out-of-pocket costs or have unmet health care needs due to costs [5, 8–10]. In return, medical costs have substantial adverse effects on AYA cancer patients as they may be more likely to borrow money, go into debt, and file for bankruptcy after a cancer diagnosis than patients diagnosed at older ages [11, 12]. As a result, many AYAs with cancer experience an elevated risk of financial toxicity (i.e., high out-of-pocket cost induced financial burden and distress) [13].

Health insurance literacy is defined as a patient’s ability to make informed decisions about choosing and using health insurance [14]. Emerging literature suggests that health insurance literacy is a key factor to enable the effective use of health care [15]. Studies have shown that even highly educated AYAs have poor health insurance literacy and need support to understand their medical bills and how to make payments [6, 16]. Importantly, a lack of understanding of insurance terms and coverage types may lead AYAs to avoid services that are exempt from cost-sharing, or to receive care that leads to unexpected costs [17]. Yet, few randomized controlled trials have tested the efficacy of insurance education to improve outcomes for cancer patients and survivors [18].

Due to the complexity of the US health care system and AYAs’ low health insurance literacy, evidence-based interventions are needed to help AYA cancer patients understand and manage their insurance, overcome health care barriers, and address health care costs during cancer treatment. Patient navigators (PNs) can help improve AYA cancer patients’ insurance literacy. PNs are proactive advocates who provide logistic and emotional support to promote patients’ access to timely care. Commonly used to address cancer disparities and negative health outcomes among vulnerable populations, patient navigation [1] includes a patient-centered focus on overcoming barriers to care, [2] aims to reduce delays in accessing care, [3] targets a defined set of health services, and [4] provides a defined endpoint when services are complete [19–21]. While financial and/or insurance navigation is a component of a few PN programs, to our knowledge, none offer tailored education to improve health insurance literacy. Additionally, even fewer are designed to address the complex, age-specific needs of AYAs.

### Objectives {7}

The overall objective of HIAYA CHAT (Huntsman Intermountain Adolescent and Young Adult Cancer Care Program ‘Let’s chat about health insurance’) is to identify whether a virtual health insurance education delivered by a PN targeted to AYAs with cancer is feasible and acceptable and shows preliminary efficacy in improving health insurance literacy and financial toxicity. HIAYA CHAT is designed in conjunction with the HINT-S program (Health Insurance Navigation Tools) that was created for long-term survivors of childhood cancer. These interventions were designed with several needs in mind: (1) The National Comprehensive Cancer Network (NCCN) Clinical Practice Guidelines in Oncology strongly recommend health insurance education, including information on sources of coverage (e.g., Marketplace plans, Medicaid) and key elements of coverage, such as copays, for young cancer patients [22]. (2) AYA cancer survivors acknowledge the need for information regarding treatment follow-up together with education about insurance and costs of care in survivorship [5, 23]. (3) Improving health insurance literacy could help young adults with little experience managing health care obtain appropriate health care.

**Fig. 1:**
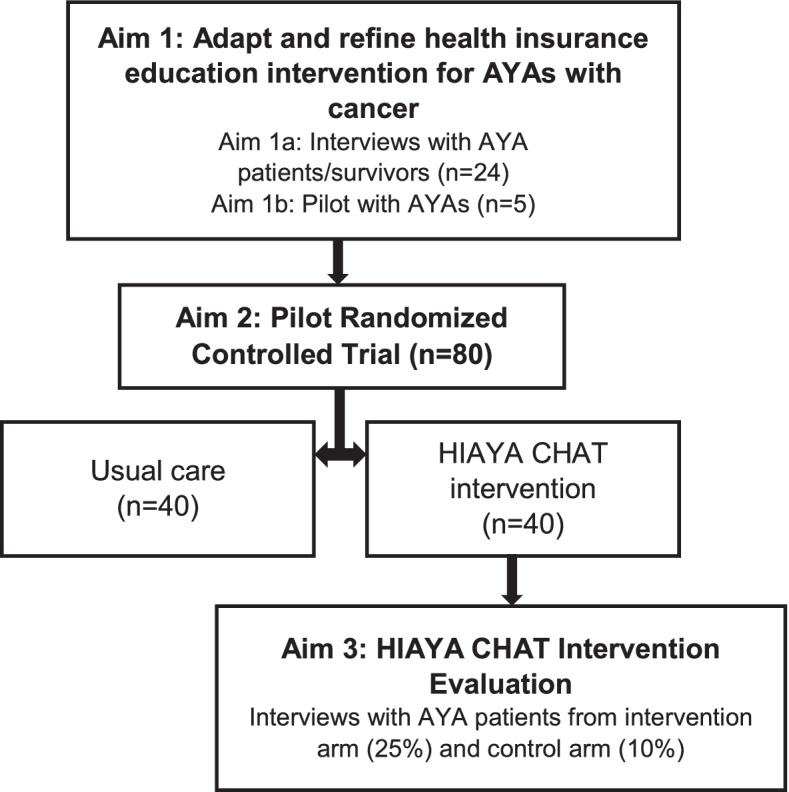
Aims outline

As seen in Fig. 1 titled “Aims outline,” there are three aims to the HIAYA CHAT study. Aim 1 focused on the adaptation of HIAYA CHAT through surveys, interviews, and pilot intervention sessions with AYA patients and survivors (Vaca Lopez PL, Warner EL, Waters AR, Mann K, Anderson JS, Ray N, et al.: Adaption and development of a health insurance education program fro adolescent and young adult cancer patients, submitted) [17]. In this manuscript, we provide an overview of Aim 2 to assess the feasibility and acceptability of the virtual pilot randomized controlled trial along with preliminary efficacy. Aim 3 will consist of interviews with control and intervention participants to evaluate participant satisfaction with the HIAYA CHAT intervention and to record recommendations for modifications on delivery and intervention content.

### Trial design {8}

**Fig. 2:**
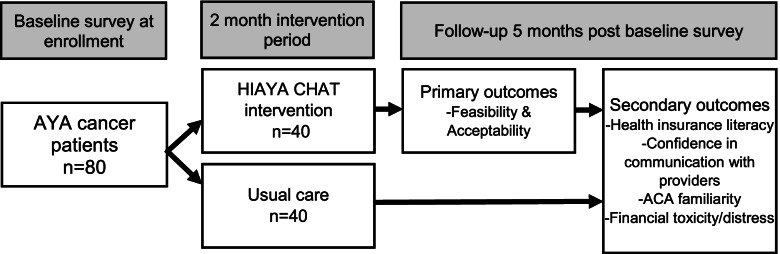
RCT design

This pilot study is a two-arm randomized controlled trial (RCT) designed to test the feasibility and acceptability of the HIAYA CHAT intervention (Fig. 2, “RCT design”). The protocol was developed in accordance with the Standard Protocol Items Recommendations for Interventional Trials Statement. It was reviewed and approved by the University of Utah and Intermountain Healthcare Institutional Review Boards.

**Fig. 3:**
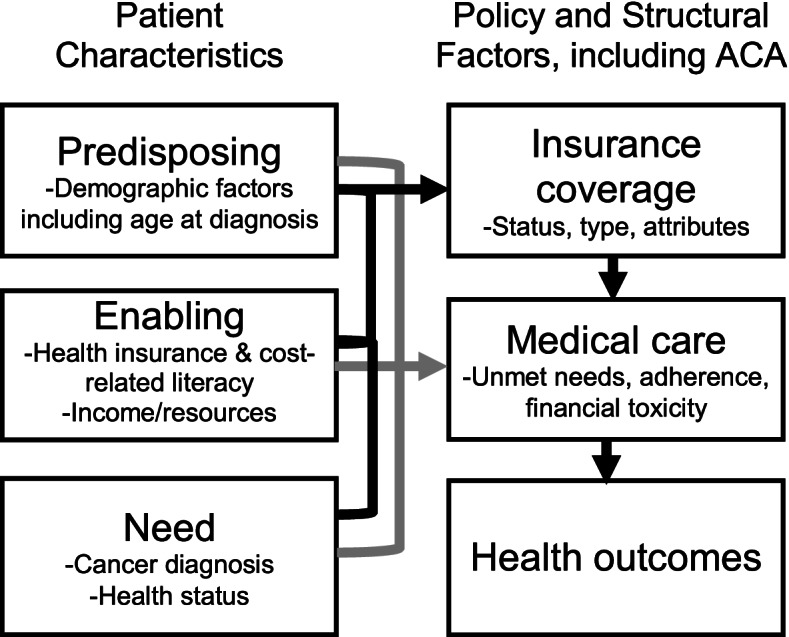
Conceptual framework

This research is founded on Levy and Meltzer’s theoretical model and Andersen’s Behavioral Model of Health Services Utilization. Levy and Meltzer’s model describes the relationship between health insurance and health. Andersen’s Behavioral Model of Health Services Utilization posits that health care use is influenced by predisposing (e.g., age, income), enabling (e.g., insurance literacy), and need factors (e.g., cancer diagnosis) [24]. As conceptualized in our trial (Fig. 3, “Conceptual framework”), predisposing factors can directly and indirectly influence individuals’ insurance status and plan characteristics, thereby influencing their use of medical care and financial toxicity. Enabling factors, such as health insurance literacy, can also directly impact coverage as well as impact the use of medical care. At the same time, the model acknowledges that insurance coverage and characteristics are influenced by trends in the larger health policy context, such as the implementation of the Patient Protection and Affordable Care Act (ACA). Thus, under the premise of these models, improving health insurance literacy can empower AYA cancer patients to access needed care and utilize their insurance to the fullest extent, ultimately improving health outcomes.

Furthermore, HIAYA CHAT utilizes motivational interviewing (MI) in the context of the care management model. Throughout this process, the study PN uses MI techniques to build and maintain self-confidence and self-efficacy as well as encourage learning and advocacy in regard to health insurance. The care management model includes (1) identifying eligible individuals, (2) identifying individual barriers to care, (3) developing an individual plan to address barriers, and (4) tracking whether barriers are overcome.

## Methods: participants, interventions, and outcomes

### Study setting {9}

The Huntsman Intermountain Adolescent and Young Adult Cancer Care Program (HIAYA) is a joint effort between the two largest cancer care providers in Utah and surrounding states: Huntsman Cancer Hospital/Huntsman Cancer Institute (HCH/HCI) at the University of Utah (U of U), the only NCI-designated Comprehensive Cancer Center in the five-state Mountain West (an area that includes Utah, Idaho, Montana, Wyoming, and Nevada), and Intermountain Healthcare (IH), a system of 23 hospitals in Utah and Idaho. Established in 2016, HIAYA supports two PNs to help guide AYA cancer patients through their care. HIAYA CHAT was funded to run through the HIAYA program. Thus, we focus recruitment on the HIAYA locations in the Salt Lake City metropolitan area where the majority of AYAs are treated for cancer: Primary Children’s Hospital (PCH) and Intermountain Medical Center (IMC), which are both of the IH system, and HCH/HCI at the U of U.

### Eligibility criteria {10}

Eligible patients, from the three hospitals included in the study, are identified according to the inclusion and exclusion criteria described in Table 1. As this is a pilot trial, the anticipated recruitment goal was 80 patients.

**Table 1:** Eligibility criteria

Inclusion criteria	Exclusion criteria
Patients are: ♦ Within 1 year of their cancer diagnosis	Patients are: ♦ Unable to perform informed consent
♦ Between the ages of 17 and 39 when diagnosed with their first cancer	♦ Unable to read, speak or understand English
♦ Receiving cancer treatment at: ○ Primary Children’s Hospital ○ Intermountain Medical Center ○ Huntsman Cancer Hospital or Huntsman Cancer Institute	♦ Currently uninsured
♦ Able to read, speak and understand English	
♦ Able to provide informed consent	

### Who will take informed consent? {26a}

Once eligibility is established, patients are contacted by a member of the research team initially through either email or in-person at an oncology clinic. Follow-up attempts are made through phone calls, text messages, or further emails. Three members of the research team are responsible for and trained in obtaining informed consent, including two study coordinators and a research assistant. If patients are reached virtually, they can consent via REDCap by reading the consent form and signing it electronically. The research team’s contact information is published in several places (e.g., recruitment emails, on the consent form) in case patients wish to reach out with questions. Patients who are approached in the clinic can discuss any questions they have with the research staff.

All participants are informed that participation in this study is voluntary and that declining to participate will in no way affect their medical care, relationship with their providers, or PN. Furthermore, potential participants are informed about the purpose of the study, potential risks and benefits, and compensation for participation (up to $60 per participant).

### Additional consent provisions for collection and use of participant data and biological specimens {26b}

No biological specimens were collected for this study. All participant data being used was clearly explained in the consent form.

## Interventions

### Explanation for the choice of comparators {6b}

Despite increasing literature that guidance on insurance and the health care system is needed to help cancer patients’ access care and manage costs, few navigation programs have structured education on these topics. Thus, usual navigation care as the comparator is justified. Usual navigation care in HIAYA typically focuses on health education, work and/or school concerns, psychosocial support, fertility concerns, finances, and insurance. While there may be some discussion of insurance between navigators and patients, these discussions are focused on overcoming obstacles rather than education on terminology and protections; the navigators track these discussions, and this information will be considered in the interpretation of the study findings. The comparison group receives usual navigation care from different patient navigators (the HIAYA PNs) than the interventionist navigator (study PN).

### Intervention description {11a}

The HIAYA CHAT intervention was designed to provide a supportive, individualized approach which includes psychoeducation with a focus on improving health insurance and cost-related literacy while overcoming barriers. Participants in the intervention arm receive 4 bi-weekly 30–45-min videoconferencing sessions over a 2-month time period. The intervention arm participants also receive usual navigation care services as needed.

All health insurance education materials were created by the research team which includes health services researchers who study health insurance and financial toxicity, clinical psychologists, PNs, a health educator, and oncologists. Materials were then critically reviewed and edited by a doctoral-trained health educator who manages the local Cancer Learning Center and is the Associate Director of Education. The control arm receives usual navigation care that consists of standard patient navigation appointments that do not include education on insurance. When the HIAYA PN first meet with AYA patients, they discuss the patient’s needs and ask about any potential concerns related to health education, work and/or school, psychosocial support, fertility concerns, finances, and insurance.

Session 1 of the HIAYA CHAT intervention includes reviewing foundational insurance terms and concepts (Table 2). For example, some of the terminology taught is “policy holder,” “premium,” and “preauthorization.” A major concept taught and shown through visuals in session 1 is how a patient’s co-pay, deductible, co-insurance, and out-of-pocket max all interact and affect each other. Session 2 was developed to be personalized to the patient’s own health insurance plan by asking participants to bring their own health insurance card and summary of benefits and coverage (SBC) to the session. In this session, the PN introduces the different health insurance types (HMO, PPO, EPO, and POS), reviews more foundational insurance terms, and then presents what information is on a health insurance card, a SBC, health bill example, and an explanation of benefits (EOB) letter. Generic examples of these documents are available in the session 2 materials in case the patient is not able to bring their own for any reason. Session 3 provides information on current insurance policies (i.e., the ACA), laws, and the internal and external appeals process. Additionally, this session introduces training and support on how to navigate resources through their hospital or via patient advocate foundations. Session 4 provides in-depth information on cost-sharing components of insurance and strategies for helping participants to estimate costs of care, including training on how to use assertive communication when having cost-of-care conversations with medical providers. At the conclusion of session 4, a list of local and national financial resources are discussed including housing, utilities, food, transportation/gas, cell phone, clothing, childcare expenses assistance programs, and cancer grants. Once the sessions are complete, the study PN reminds participants to connect with the HIAYA PN for further navigation needs.

**Table 2:** HIAYA CHAT intervention modules to improve health insurance literacy

**Session 1: insurance terms and concepts**	
Informational: define and explain basic insurance terms, concepts, and insurance types	
Assessment: participant’s understanding of financial concepts related to cancer care	
Overcoming barriers: reviewing current copay, deductible, and out-of-pocket maximum	
Resource provision: guidance on how health insurance works within the medical realm	
**Session 2: your health insurance plan**	
Informational: learning about one’s own plan and benefits policies	
Assessment: utilizing one’s plan, accessing benefits, and navigating resources	
Overcoming barriers: exploring what one is entitled to in a plan and how to optimize benefits	
Resource provision: worksheet called “Know Your Health Insurance”	
**Session 3: healthcare laws and appeals**	
Informational: learning about healthcare laws (ACA, COBRA, ADA, FMLA) and the appeals process	
Assessment: utilizing health insurance law protections	
Assessment: utilizing internal and external appeals process	
Overcoming barriers: exploring when and how to appeal a health insurance claim	
Resource provision: connecting with triage cancer information and patient advocacy foundations	
**Session 4: cost controlling strategies**	
Informational: understanding other cost-sharing mechanisms	
Assessment: participant’s understanding of financial concepts related to cancer care	
Assessment: estimating the costs of obtaining a needed service	
Overcoming barriers: strategies for decreasing out-of-pocket costs	
Overcoming barriers: options to pursue if a needed service, medication, or provider is not covered by insurance (e.g., financial counselors), difficult to access, or cost prohibitive	
Overcoming barriers: promoting communication with medical providers about costs	
Resource provision: connecting with state or health plan-based price transparency resources	

### Criteria for discontinuing or modifying allocated interventions {11b}

If a randomized participant asks to withdraw from the intervention sessions, they are asked why they wish to discontinue to inform our feasibility findings. All participants receive the 5-month follow-up survey, including those who ask to discontinue the intervention sessions, unless participants ask to stop receiving communication and withdraw from the research study. During data analysis, we will examine factors that may be different between individuals who complete the HIAYA CHAT intervention and individuals who do not, as this is important to understanding the feasibility.

### Strategies to improve adherence to interventions {11c}

A notable methodological consideration pertaining to the proposed research is protection against attrition. In our previous work, we have learned that individuals are best retained in studies that allow for (1) intervention sessions delivered virtually through videoconference with maximal flexibility, (2) multiple modality options to conduct follow-up assessment and follow-up assessment reminders, and (3) remuneration for surveys completed.

Research team members who lead recruitment have received training in participant engagement from the study coordinator. Trainings consisted of didactics and role plays. We also provide training on effective patient tracking systems (call attempts) and retention strategies (survey reminders, follow-up) and best practices on documentation. We ensure that interested participants receive a thorough explanation of the study options, requirements, and follow-up procedures. Study staff will emphasize responsibilities as a research participant, in particular the intervention nature of the study, reiterate confidentiality, and develop good rapport.

To increase the study PN adherence to intervention materials, we created two tools to document intervention fidelity: (1) PDF fidelity sheets and (2) audio recordings of each intervention session. A PDF fidelity sheet was created for all four sessions and includes every definition and concept the study PN needs to review with participants. The fidelity sheet is filled out by the study PN for every session with every participant. These fidelity sheets and audio recordings are reviewed by the research team to make sure each participant is being taught all the health insurance concepts described in sessions 1–4.

### Relevant concomitant care permitted or prohibited during the trial {11d}

Relevant concomitant care includes any discussions and resources given between the HIAYA PN and the participants themselves. Everyone in the RCT is given usual care and has access to a HIAYA PN. All HIAYA PNs are familiar with the study protocol and have agreed to audio record or document any conversations they have about health insurance and finances with participants in this study, including control arm participants. When in-depth questions concerning health insurance and finances are asked of the HIAYA PNs, they do their best to help answer the question and also give the participants resources (e.g., Triage Cancer links, contact information of hospital financial counselor), which is considered usual care.

### Provisions for post-trial care {30}

One advantage of conducting the HIAYA CHAT study within the HIAYA program is the ability to offer post-trial care. For those in the intervention condition, their care is transferred from the study PN to the HIAYA PNs directly after session 4 concludes, for any further questions and future issues. The control participants are always connected to the HIAYA PNs throughout and beyond the study. All control participants will receive the intervention materials at the conclusion of the study through e-mail.

### Outcomes {12}

Our primary outcomes are feasibility and acceptability, defined as follows: (1) feasibility: number of eligible enrolled and number sessions completed and (2) acceptability: completion of a 5-point scales of satisfaction with the HIAYA CHAT intervention (e.g., “To what extent did the HIAYA CHAT intervention meet your needs?” “Did you get the kind of insurance assistance that you wanted?” “How helpful has this intervention been for you?”) and the validated Patient Satisfaction with Interpersonal Relationship with Navigator tool [25, 26]. We will examine the percentage of participants reporting satisfaction with these responses. As this is direct patient-reported satisfaction, this should be a good indicator of future feasibility of this intervention.

Our secondary outcomes assess preliminary efficacy: (1) health insurance literacy as assessed by the Health Insurance Literacy Measure (HILM), including confidence discussing health care costs with providers [14, 27]; (2) familiarity with ACA policies [28]; and (3) financial distress related to medical costs, including the COmprehensive Score for financial Toxicity (COST) [29, 30]. These questions and measures will be given in both baseline and follow-up questionnaires. For HILM and COST, we will examine difference in scores between the two time points, examining differences by demographic factors as well as receipt of the intervention. For questions surrounding familiarity with the ACA policies, questions will also be given in both the baseline and follow-up questionnaires. We will examine the percentages reporting familiarity at both timelines.

Improvement in health insurance literacy and decreases in financial toxicity are the ultimate goals of providing a health insurance education to participants. Examining differences between these measures, before and after receiving the intervention, should give a good indication of the effectiveness of this study. We will also be able to measure if participants were able to retain information provided to them through the intervention about the ACA policies.

### Participant timeline {13}

Participants are asked to take part in the study for approximately 5 months, which includes the baseline survey, participation in the intervention or control arms, and a follow-up survey. Some participants may also be asked to complete an interview after these activities have been completed with a member of the research team.

**Fig. 4:**
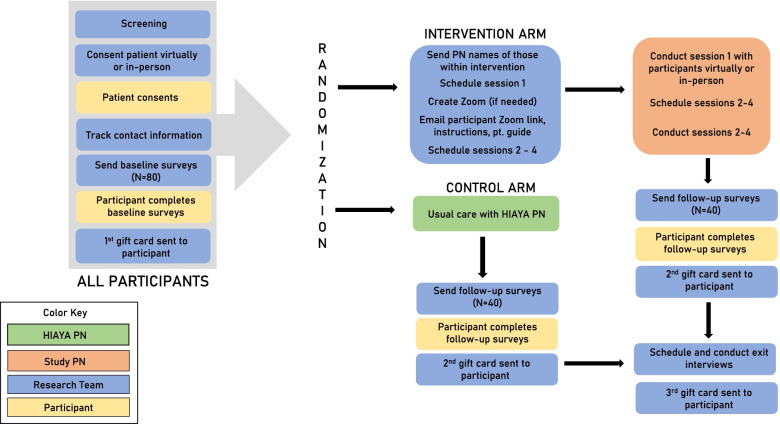
Participant timeline

Each patient consents into the study and then immediately takes the baseline survey online, which is created to take about 15–20 min to complete. At this point, the research team randomizes the participant into either the intervention arm or the control arm. Participants are then notified, through email, which arm they will be participating in and what next steps will occur as they continue in the study. For the control arm, the next steps include taking the follow-up survey 5 months after baseline survey completion and then 10% of the control arm is invited to participate in a short phone exit interview. For the intervention arm following randomization, they are contacted by the study PN to schedule a time for their first HIAYA CHAT session. At the conclusion of each session, the following session can be scheduled with the PN up until the end of session 4. Each session is generally held 1 to 2 weeks apart. Intervention participants also receive the follow-up survey 5 months after completing their baseline survey and then 25% of the intervention arm is invited to participate in a short phone exit interview. Electronic gift cards ($20 each) are given after each survey is complete and at the conclusion of the exit interview, when applicable. This timeline can be seen visually in Fig. 4 titled “Participant timeline.”

### Sample size {14}

Our primary outcomes of interest are the feasibility and acceptability of the HIAYA CHAT intervention. We intend to enroll a total of 80 participants and randomize 40 participants per arm, which we have selected to ensure the evaluation of feasibility and to explore meaningful differences in the outcomes. We expect at least 72 participants to complete the 5-month follow-up survey (10% attrition rate). For financial toxicity, our preliminary data show that for the 11-item COST score, average scores at enrollment in the navigation program for AYAs range from 25.79 (standard deviation (SD) = 10.11) for younger AYAs to 18.22 (SD = 10.81) for older AYAs [30]. Lower COST scores indicate greater financial distress. We expect to have >80% power to detect differences in the mean improvement in the COST score between the intervention and control arms which differ by 0.67 SD based for our target *N* = 72 completing the RCT.

### Recruitment {15}

For recruitment, we are focusing our efforts on three hospitals: PCH, IMC, and HCH/HCI. The research team has several avenues to screen for new participants: (1) we monitor patient enrollment into the HIAYA program through the registries, (2) we screen clinic schedules for eligible participants who are not currently enrolled in HIAYA and approach them in during outpatient clinic visits, (3) we screen through responses to advertisements on the HIAYA social media accounts, and lastly (4) we ask clinicians who regularly see AYA cancer patients for referrals. Our goal is to enroll 6–7 participants each month for a total of 80 participants. In 2017, there were 569 newly diagnosed age-eligible patients treated at HCH/HCI, and approximately 270 newly diagnosed age-eligible patients were treated in total at our three Intermountain clinics, ensuring that we will have an adequate number of potential participants to approach for recruitment.

We primarily recruit participants through email contact, text messages, and phone calls as recruitment launched during the first year of the COVID-19 pandemic. However, more recently as restrictions were altered, we sometimes approach potential participants in person when they present to clinic.

## Assignment of interventions: allocation

### Sequence generation {16a}

Randomization is stratified by age at survey (18–25; 26–39 years) and treatment site (PCH, IMC, HCH/HCI). Random allocations within strata are computer generated through the survey software REDCap; the lead statistician on this research team coded the REDCap project to be able to randomize the participants once age and treatment site were entered into specific fields. Once a participant consented into the study and finished the baseline survey, they are randomized by the study coordinator who would log into REDCap, type in age and treatment site, then click the randomization button. After randomization occurs, the participant would be emailed and told which arm they had been randomized into with tailored directions for the next steps of the study.

### Concealment mechanism {16b}

After a participant has consented and taken the baseline survey, they are randomized according to pre-defined randomization schedules with randomization concealment and access via REDCap. A study coordinator inputs the patient identifier, site of recruitment, and age into the algorithm and receives an output informing the coordinator of the participant’s randomization arm.

### Implementation {16c}

The lead statistician created and input the allocation sequence into the HCI REDCap software. Two study coordinators led the screening, recruitment, data entry, randomization, and notifying participants if they were part of the intervention arm or the control arm.

## Assignment of interventions: blinding

### Who will be blinded {17a}

The recruitment side of the research team (study coordinators and research assistant) and the PI are not blinded. The statistician and data manager are blinded to the treatment allocation, until the conclusion of data analysis, as not to bias the results.

Neither the statistician nor the data manager, who will perform post-trial analysis, will have direct access to the survey information in REDcap; instead, they will receive that data from the study coordinator which will not include information on participant’s randomization arm. When analysis to compare the two randomization arms is done, study coordinators will send a file which will continue to conceal arms by using group A and group B instead of actual randomization arm names.

### Procedure for unblinding if needed {17b}

The PI, study coordinators, and research assistant are unblinded at the time of randomization. This is necessary so all other study procedures can take place. The statistician and data manager will only be unblinded when all data analysis is complete.

## Data collection and management

### Plans for assessment and collection of outcomes {18a}

All baseline and follow-up survey data are captured and saved within the REDCap software until the completion of the RCT. Survey items are listed in Table 3 along with the source of where the questions were taken from. Demographics will be gathered through the baseline survey. Feasibility is being measured by the number of sessions completed which is documented in REDCap by the study PN. For acceptability, the 5-point scales of satisfaction are on the follow-up survey as well as the Patient Satisfaction with Interpersonal Relationship with Navigator tool [25, 26]. For our secondary outcomes, the HILM [14], COST, and questions asking familiarity with ACA policies are all on both the baseline and follow-up surveys [29, 30].

**Table 3:** Survey domains

Items shown in bold are primary or secondary outcomes		Source	BL	FU	Session
Demographics	Age, date of diagnosis, gender, sexual orientation, race/ethnicity		X		
Demographics repeated	Treatment status, partnership/marital status, education level, zip code, current living situation		X	X	
Enabling characteristics	**Familiarity with ACA policies**	CCSS insurance survey	X	X	Session 3
	**Health insurance literacy** 1. Understanding of terms (e.g., premium, deductible, co-payments, co-insurance, out-of-pocket maximum, annual limits) 2. Confidence in using insurance plans	Commonwealth Fund Annual Insurance Survey Health Insurance Literacy Measure	X	X	Session 1
	**Cost-related literacy:** comfort/confidence talking about medical costs with providers, understanding of financial concepts of care	CCSS insurance survey	X	X	Session 4
	Household and personal income, employment status		X	X	All sessions
	Cancer diagnosis, age at and years since diagnosis, treatment		X	X	Session 1
Need	Health status		X		Session 1
	Insurance status, type, duration	CCSS insurance survey	X	X	All sessions
Insurance coverage	Denial or difficulty obtaining coverage because of health history (within past 2 years)	CCSS insurance survey	X	X	Session 3
	Not taking a new job in order to keep health insurance in past year		X	X	Session 3
	Trouble finding a provider who accepts insurance/getting an appointment as needed		X	X	Session 3
Underinsurance and costs	Unmet health care need due to cost; provider visits past year; out of pocket medical costs > 10% of income	CCSS insurance survey	X	X	Session 4
	Worry/problems about medical costs (e.g., problems paying bills)	COmprehensive Score for financial Toxicity	X	X	Session 4
	**Financial distress/toxicity**		X	X	Session 4
	Policy holder (e.g., self, spouse, parent) and source		X	X	Session 1
	Plan source: employer-sponsored, direct purchase (exchange or outside), Medicaid, Medicare	CCSS insurance survey	X	X	Session 2
Coverage-related variables	Type of plan: high-deductible plan; narrow network plan	CCSS insurance survey	X	X	Session 2
	Being forced to switch plans because of cancelation (in past year)		X	X	Session 2, 3
	Rating of current plan		X	X	Sessions 2 and 3
**Satisfaction with navigation**		Patient Satisfaction with Interpersonal Relationship with Navigator		X	

### Plans to promote participant retention and complete follow-up {18b}

We are using several strategies to promote retention. Participant contact information is obtained in multiple avenues (phone numbers, email addresses, and mailing addresses) and saved in a password-protected and secure server at HCI. In addition, we have designed the HIAYA CHAT intervention to be delivered via videoconference or in-person, meaning that the sessions can occur at the convenience of the participant. Similarly, participants in both the intervention and control arms of the trial complete surveys that are either done in person during clinic appointments or over an e-mailed REDCap link, thus reducing their burden. All surveys are created using design principles of Dillman et al. to ensure survey is appropriately and consistently designed for multiple modes (e.g., on home computer/laptop, in-person via iPad) and to minimize participant burden. We use good visual layout, question-writing techniques, and design principles to make the surveys easy to navigate. Steps to achieving these goals include limiting survey length and complexity, employing skip patterns, ordering and grouping similar questions with respect to content and response sets, and using standard visual design principles (e.g., size, font, color, and visual element location). In addition, participants are provided with a $20 gift card as a thank you for their participation at each survey time point (baseline and 5-month follow-up surveys for $40 total) and an additional $20 for those who complete the phone exit interview for a total of $60.

### Data management {19}

All surveys are taken online through the REDCap system, either on the participants own time or with a study coordinator. To check for any data entry mistakes, research assistants look through the records stored in REDCap weekly to check for any irregularities. Careful monitoring of the recruitment, enrollment, retention, adverse events, and study procedures helps to protect the safety of study participants, the quality of data, and the integrity of the trial. As part of the safety plan, the lead study coordinator reviews each participant’s record to ensure that appropriate mechanisms to protect the safety of study participants are being followed, that protocol requirements are adhered to, and that data are accurate, complete, and secure. Participant records include consent forms, data flow forms, inclusion/exclusion screening forms, questionnaires, and adverse event logs. Only the study staff will have access to the audio files from the interviews. We keep the audio files on secure, password-protected computers and servers maintained by HCI. Audio files will not be used in public presentation of research results.

Physical data will be maintained for 3 years after the completion of the study. At that point, all physical surveys or information will be digitalized and stored on password-protected computers in encrypted files.

### Confidentiality {27}

All study personnel have completed Human Subjects Protection Training through the Collaborative Institutional Training Initiative (www.citiprogram.org). A key component of the training is a review of privacy measures of HIPAA and HIPAA policies relevant to research, including with children and their families, to protect the confidentiality of patients and research subjects. All study data and randomization allocation tables reside at the HCI’s encrypted network, which is a HIPAA-compliant protected environment.

All measures use numerical codes (unrelated to the patient’s Protected Health Information) to identify participants and names will not appear on any of the collected data. The data file linking names and the unique participant identifier are accessible only to the PI and research coordinators in order to monitor and coordinate retention and follow-up with participants. Signed copies of the consent/assent forms are kept in a locked file cabinet in the locked office of the PI and will be separated from data provided by participants. All data will be controlled by the PI and the trained research coordinators.

The research team members make every effort to prevent the occurrence of breach of confidentiality. All computers used to access data are encrypted and password protected. All REDCap data are stored on a server that is HIPAA compliant (21-CFR-11) and has the strongest protection afforded by the University of Utah and HCI. Only study personnel have access to the data and all data are encrypted with a key as it is written in the backup tape. Without the key, the data on the tape are unreadable. These methods of protecting participant confidentiality is required by the University of Utah’s and will continue to be used for the remainder of this study.

### Plans for collection, laboratory evaluation, and storage of biological specimens for genetic of molecular analysis in this trial/future use {33}

Not applicable. No biological specimens were collected for this study.

## Statistical methods

### Statistical methods for primary and secondary outcomes {20a}

We will use descriptive statistics to report on the outcome measures. We will run descriptive statistics to examine arm differences at baseline; any imbalanced covariates will be included as adjustment variables. We will use chi-squared and independent sample *t*-tests to compare end-of-treatment changes in preliminary efficacy outcomes between the two arms. We will compare baseline/end-of-treatment, within arms, with paired *t*-tests for continuous outcomes and McNemar’s tests for categorical outcomes. In addition, we will use bivariate statistics to examine demographic and cancer-related factors (type of diagnosis, age at diagnosis, treatment) and health status with feasibility, acceptability, and preliminary efficacy outcomes.

Multivariate analyses, adjusting for imbalanced covariates, will be conducted in the context of linear or logistic models, potentially with random effects for patient, as appropriate. We will test for differences by sex, age, and race/ethnicity in the effects. We will also conduct analyses to determine moderators of the intervention effects. Tests of interactions between covariates (moderation analyses) will be conducted in the context of both the unadjusted and adjusted versions of the regression models with effects for both the covariate of interest and the treatment arm, as well as additional covariate by covariate interaction terms.

We will also conduct analyses to determine potential moderators of the intervention effects. Tests of interactions between covariates (moderation analyses) will be conducted in the context of both the unadjusted and adjusted versions of the above generalized estimating equations models with effects for both the covariate of interest and the treatment arm, as well as additional covariate by covariate interaction terms. For example, we will explore whether differences exist in the feasibility and acceptability of the HIAYA CHAT interventions for rural participants, who often face access-related barriers to health care. As the Mountain West (Utah, Idaho, Montana, Nevada, and Wyoming) is a large geographic area with rural communities, many AYAs drive long distances into Salt Lake City, UT, for their cancer care [31].

### Interim analyses {21b}

Not applicable. No interim analyses are needed or planned for our study.

### Methods for additional analyses (e.g., subgroup analyses) {20b}

We examine differences in our outcomes of interest for younger patients (diagnosed between ages 18 and 25) and older patients (diagnosed between ages 26 and 39).

### Methods in analysis to handle protocol non-adherence and any statistical methods to handle missing data {20c}

For missing data that is demographic in nature and is also collected in the electronic medical record (EMR), we will use EMR data to supplement missing responses. For outcome measures such as COST and HILM, which consist of combining responses across multiple questions to get a measure score, participants missing values would result in potentially artificially lower scores which indicate higher financial toxicity and lower health insurance literacy. Without a complete measure, we cannot accurately assess the impact of the HIAYA CHAT intervention and as such, participants who do not complete all questions associated with these measures will not be included in the analysis for our second objective.

As for our analysis surrounding acceptability and efficacy, we will include individuals with missing responses as that is an important factor for accurate conclusions.

### Plans to give access to the full protocol, participant-level data, and statistical code {31c}

Trial protocol and statistical code will be available per author contact. Participant data will be provided with appropriate IRB approvals.

## Oversight and monitoring

### Composition of the coordinating center and trial steering committee {5d}

The trial coordinating center is overseen by Dr. Kirchhoff with program management by Karely Mann, MS. Further support is provided by Dr. Park (PhD, MPH) and Dr. Perez (PhD). Ms. Mann has over 6 years of experience running research studies and conducts the day-to-day study operations including working with the study PN and working research assistants who are responsible for participant recruitment. Ms. Mann is supported by Perla Vaca Lopez (BS) who is focused on recruitment. Dr. Ben Haaland (PhD) is our lead statistician with support from data manager Ms. Heydon Kaddas (MPH).

### Composition of the data monitoring committee, its role, and reporting structure {21a}

We estimate the risk level associated with this study to be minimal. We have established a data and safety monitoring plan (DSMP) to ensure the safety of participants. These activities include (1) review of screening results and any other available data at weekly research meetings between the PI and research staff, as well as any other relevant investigative team members; (2) a quarterly review of data safety and enrollment by the research team, including the PI, the study coordinator, and relevant investigative team members; and (3) an annual review by the University of Utah Institutional Review Board (IRB).

### Adverse event reporting and harms {22}

Anticipated adverse events (AEs) include participant distress related to the intervention being tested and loss of participant confidentiality. If an anticipated AE occurs, the PI will be immediately notified, and a note will be entered into the participant’s file. The PI and the investigative team will be responsible for evaluating each AE and determining attribution as well as the impact of the AE on the risk/benefit ratio.

All unanticipated and/or serious AEs will be reported to the IRB within 24 h of occurrence. The IRB and the PI and the investigative team will be responsible for determining whether modifications to the protocol and consent/assent forms are required. If a determination is made that participants are found to be exposed to excessive risks in relation to anticipated benefits, the trial will be immediately suspended. The trial will not resume until modifications are made that are deemed to result in an acceptable risk/benefit ratio by the PI/investigative team and IRB. Aggregate reports of AEs will be prepared as required and forwarded to the IRB for review.

### Frequency and plans for auditing trial conduct {23}

In addition to fidelity checks mentioned above, the research team does regular internal audits on gift card distribution logs. If any issues arise concerning gift cards, that information is reported to the PI during weekly meetings. HCI’s Cancer Control and Population Sciences department supports an auditor that is external to the research team. These HCI audits occur randomly and the auditor will communicate with the research team if HIAYA CHAT is chosen to be audited.

### Plans for communicating important protocol amendments to relevant parties (e.g., trial participants, ethical committees) {25}

Any major protocol amendments will be communicated with the University of Utah and Intermountain Healthcare’s IRBs immediately through an online report form. Once the report is reviewed, the IRB communicates back to the research team if they need to communicate with trial participants or not. Despite this being a low-risk trial, the research team is dedicated to communicating any alterations that need to be mentioned to participants through email.

## Dissemination plans {31a}

The PI is responsible for ensuring that this clinical trial is registered and that result information is submitted in a timely manner to ClinicalTrials.gov. The informed consent documents for this trial include a specific statement relating to the posting of clinical trial information at ClinicalTrials.gov.

Our team is dedicated to dissemination of research findings to patients, providers, and policy-makers and has a strong history of scientific publications. Publications from this research will be made available to the public through the National Library of Medicine PubMed Central website within 1 year after the date of publication. We will publish findings related to each of the study aims. Publications so far included results from the Aim 1 interviews [17]. Future publications will include the Aim 2 feasibility, acceptibility, and intervention-associated health insurance literacy and financial toxicity improvements. We will present the study design and findings at national and/or international scientific meetings to reach clinicians and researchers in the areas of health insurance, cancer survivorship, AYA cancer, and behavioral sciences. We will present on our study findings and processes (e.g., recruitment and screening) at professional meetings (e.g., Southwest Oncology Group, ASCO Quality Care Symposium).

## Discussion

Health insurance education interventions targeted at AYA cancer patients hold great potential to help this growing population understand and manage their health insurance, overcome health care barriers, and help improve health insurance literacy and literacy regarding managing health care costs. A key aspect of this intervention is having a PN as the interventionist, allowing for the insurance and cost-related discussions to occur in a personal (one-on-one) setting. The HIAYA CHAT trial is ongoing (recruitment is complete, but follow-up data are in collection). Additionally, because this trial is set within an AYA program that covers the Mountain West (i.e., the HIAYA Cancer Care Program), our feasibility results will be able to inform similar programs that wish to include health insurance education as a service they provide for their young cancer patients.

This trial is innovative in several different ways: (1) This is the first program designed specifically to improve AYA cancer patients’ health insurance and cost-related literacy, which could have a significant impact on treatment adherence, financial toxicity, and health outcomes for this growing population. (2) Patient navigation has been widely used in oncology clinical care, but using navigators to improve health insurance and cost-related literacy is novel. Navigation has been employed to help consumers choose an insurance plan and manage costs [32], but not to address continuing insurance needs which will arise as they go through cancer treatment and continue through survivorship. (3) Through the study PN, we are testing our HIAYA CHAT intervention through videoconferencing as an innovative means of delivering education. Given the high prevalence of virtual technology use among AYAs [33, 34] and the increased use of telehealth during the COVID-19 pandemic, delivering navigation through technology-based platforms increases flexibility and accommodates AYAs unique resource needs. This format can be adapted to be provided by navigators, social workers, and health educators at other locations in the future.

Although the COVID-19 pandemic began a few months before we planned to start recruitment, we had already created this RCT to include videoconferencing as the main delivery option. Very few changes were needed in order to make this intervention possible for AYAs during this unique time. Telehealth visits have increased dramatically since the pandemic began and may continually be accepted by patients as a “normal” delivery option for their health care needs. The HIAYA CHAT trial is timely to test the feasibility of this videoconference-based health insurance education intervention.

## Trial status

Within the ClinicalTrials.gov system, the HIAYA CHAT study is logged under the ID: NCT04448678. We have not needed to alter our study design so the protocol version number is 1. Small updates were last logged in ClinicalTrials.gov on August 8 of 2021. Recruitment for the HIAYA CHAT study began on October 21 of 2020 and recruitment to consent into the study ended on December 17, 2021. The study is still ongoing with intervention sessions finishing and follow-up surveys scheduled out until May 2022. Exit interview invitations will also be conducted until June 2022 (a participant's follow-up survey needs to be completed before they are eligible to participate in the exit interview). Typically, it is advised that study protocols are submitted well before recruitment completes, yet this study protocol was submitted once the recruitment process was complete with study tasks still ongoing. The authors decided to delay the submission of this protocol because recruitment methods were continually being updated due to the COVID-19 pandemic and the difficulty of recruiting younger AYA patients. Where we originally planned to recruit almost solely in-person, we ended up recruiting heavily through email, phone calls, text messages, and social media posts. To honestly and fully describe what recruitment required for this study protocol, we needed to delay our submission. In addition, this delay allowed for the protocol to be published in this journal in conjunction with other cancer financial hardship intervention protocols. 

## Data Availability

Specific members of the research team at HCI (Dr. Kirchhoff, Ms. Mann, Mr. Waters, Dr. Haaland, and Ms. Kaddas) will have access to the final trial dataset so that data analysis and manuscript writing can occur.

## Abbreviations

ACA: The Patient Protection and Affordable Care Act
AYA: Adolescent and young adult
COST: COmprehensive Score for financial Toxicity
COVID-19: Coronavirus disease 2019
EMR: Electronic medical record
EOB: Explanation of benefits
FMLA: Family and Medical Leave Act
HIAYA CHAT Study: Huntsman Intermountain Adolescent Young Adult and Cancer Health insurAnce Tools Study
HIAYA Program: Huntsman Intermountain Adolescent Young Adult Cancer Care Program
HILM: Health Insurance Literacy Measure
HINT-S: Health Insurance Navigation Tools
HIPAA: Health Information Portability and Accountability Act
HCH: Huntsman Cancer Hospital
HCI: Huntsman Cancer Institute
IH: Intermountain Healthcare
IMC: Intermountain Medical Center
MI: Motivational interviewing
NIH: National Institute of Health
PCH: Primary Children’s Hospital
PI: Principal investigator
PN: Patient navigator
RCT: Randomized controlled trial
SBC: Summary of benefits and coverage
U of U: University of Utah
